# Notched Teeth

**Published:** 1883-11

**Authors:** 


### Notched Teeth.

In a paper read at the Societe de Chirurgie of Paris (*Brit. Med. Jour.*, July 7, 1883), M. Magitot lately called attention to the notching and erosions of the teeth in inherited syphilis, and on the relations of this disease to rickets. He thinks that the notch is not characteristic, and states that it is never found in some races frequently affected by syphilis, such as the Japanese and Peruvians. According to Magitot, not only inherited syphilis, but also all other serious troubles of nutrition, may cause diminution in the number and size of the teeth, or decay in the period of their eruption, but never erosion. Most frequently, the latter is caused by certain nervous affections of early childhood such as infantile convulsions, especially when accompanied by general debility.

The sweeping of the Paris streets, according to the last official return, cost $1,048,600. The number of persons employed in the work is 3,016, including 820 sweepers, 2,010 auxiliary sweepers, and 186 foremen.

